# Elective induction of labor at 39 weeks among nulliparous women: The impact on maternal and neonatal risk

**DOI:** 10.1371/journal.pone.0193169

**Published:** 2018-04-25

**Authors:** Rachel G. Sinkey, Jasmin Lacevic, Tea Reljic, Iztok Hozo, Kelly S. Gibson, Anthony O. Odibo, Benjamin Djulbegovic, Charles J. Lockwood

**Affiliations:** 1 Department of Obstetrics and Gynecology, Division of Maternal-Fetal Medicine, University of Alabama at Birmingham, Birmingham, Alabama; 2 University of South Florida Health Program for Comparative Effectiveness Research, University of South Florida Morsani College of Medicine, Tampa, Florida; 3 Department of Mathematics, Indiana University Northwest, Gary, Indiana; 4 Department of Obstetrics and Gynecology, Division of Maternal-Fetal Medicine, MetroHealth Medical Center-Case Western Reserve University, Cleveland, Ohio; 5 Department of Obstetrics and Gynecology, Division of Maternal-Fetal Medicine, University of South Florida, Tampa, Florida; 6 Tampa General Hospital, Tampa, Florida; 7 Department of Supportive Care Medicine, City of Hope, Duarte, California; PreTeL, Inc, UNITED STATES

## Abstract

**Objective:**

Optimal management of pregnancies at 39 weeks gestational age is unknown. Therefore, we sought to perform a comparative effectiveness analysis of elective induction of labor (eIOL) at 39 weeks among nulliparous women with non-anomalous singleton, vertex fetuses as compared to expectant management (EM) which included IOL for medical or obstetric indications or at 41 weeks in undelivered mothers.

**Materials and methods:**

A Monte Carlo micro-simulation model was constructed modeling two mutually exclusive health states: eIOL at 39 weeks, or EM with IOL for standard medical or obstetrical indications or at 41 weeks if undelivered. Health state distribution probabilities included maternal and perinatal outcomes and were informed by a review of the literature and data derived from the Consortium of Safe Labor. Analyses investigating preferences for maternal versus infant health were performed using weighted utilities. Primary outcome was determining which management strategy posed less maternal and neonatal risk. Secondary outcomes were rates of cesarean deliveries, maternal morbidity and mortality, stillbirth, neonatal morbidity and mortality, and preferences regarding the importance of maternal and perinatal health.

**Results:**

A management strategy of eIOL at 39 weeks resulted in less maternal and neonatal risk as compared to EM with IOL at 41 weeks among undelivered patients. Cesarean section rates were higher in the EM arm (35.9% versus 13.9%, p<0.01). When analysis was performed only on patients with an unfavorable cervix, 39 week eIOL still resulted in fewer cesarean deliveries as compared to EM (8.0% versus 26.1%, p<0.01). There was no statistical difference in maternal mortality (eIOL 0% versus EM 0.01%, p = 0.32) but there was an increase in maternal morbidity among the EM arm (21.2% versus 16.5, p<0.01). There were more stillbirths (0.13% versus 0%, p<0.0003), neonatal deaths (0.25% versus 0.12%, p< 0.03), and neonatal morbidity (12.1% versus 9.4%, p<0.01) in the EM arm as compared to the eIOL arm. Preference modeling revealed that 39 week eIOL was favored over EM.

**Conclusions and relevance:**

Mathematical modeling revealed that eIOL at 39 weeks resulted in lower population risks as compared to EM with induction of labor at 41 weeks. Specifically, eIOL at 39 weeks resulted in a lower cesarean section rate, lower rates of maternal morbidity, fewer stillbirths and neonatal deaths, and lower rates of neonatal morbidity.

## Introduction

Timing of delivery is a vital component of a healthy pregnancy. An increase in morbidity and mortality exists on both ends of the gestational age at delivery spectrum. On one hand, preterm birth is the leading cause of neonatal morbidity and mortality in the United States and is associated with substantial societal and healthcare costs.[1, 2] On the other, late-term and post-term pregnancies are also associated with increased maternal, fetal and neonatal risks.[3] Because of these risks, the American College of Obstetricians and Gynecologists (ACOG) states that a provider may consider induction of labor between 41 0/7 and 41 6/7 weeks gestational age and recommends induction of labor after 42 0/7 weeks gestational age.[3]

While obstetricians generally agree on the management of late- and post-term pregnancies, uncertainty exists over the optimal timing of delivery among pregnancies between 39 and 41 weeks gestation. On one hand, elective induction of labor at 39 weeks gestational age avoids potential risks of ongoing pregnancies including preeclampsia and stillbirth. One European analysis quantified the total pregnancy loss rate at 39, 40 and 41 weeks as 1.4%, 2.4% and 2.8%, respectively.[4] Additionally, elective induction decreases the risk of macrosomia with its attendant risk of shoulder dystocia with or without permanent brachial plexus injury. Both preeclampsia and fetal macrosomia increase the risk of cesarean delivery.[5–10] However, induction of labor also carries risk. Women who undergo induction of labor have higher rates uterine hyperstimulation and Category II and III fetal heart rate tracings.[11] Additionally, nulliparous patients with an unfavorable cervix undergoing induction of labor may carry a higher rate of cesarean delivery.[12–14]

Available literature to guide clinicians is biased by retrospective data and prospective studies that are underpowered to detect differences in several clinically important outcomes.[15, 16] When empirical studies do not exist or are difficult to conduct, mathematical modeling may be the best option available to derive rational policy. Therefore, in light of this clinical equipoise, our objective was to perform a comparative effectiveness analysis of elective induction of labor at 39 weeks gestational age among nulliparous women with uncomplicated pregnancies as compared to expectant management with induction of labor for standard medical or obstetrical indications or at 41 weeks gestation in undelivered mothers.

## Materials and methods

### Model overview

**Fig 1:**
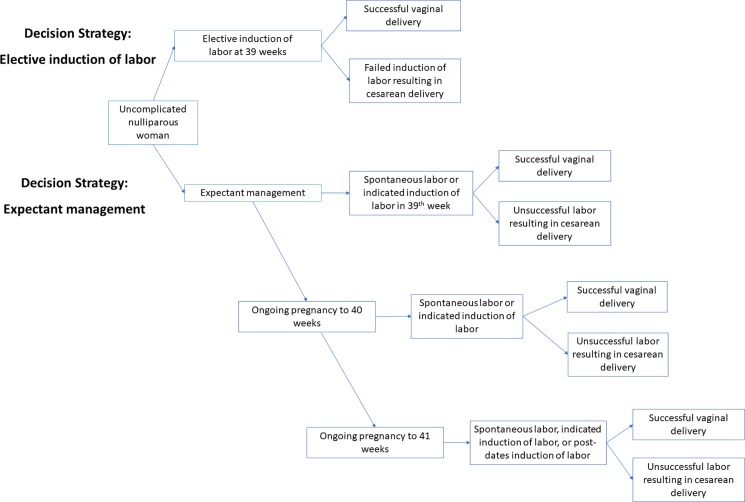
Elective induction of labor decision tree model. This model schematic demonstrates two mutually exclusive arms: elective induction (eIOL) of labor at 39 weeks or expectant management with induction of labor for medical and obstetrical indications or by 41 weeks for undelivered patients.

A Monte Carlo micro-simulation model was constructed using TreeAge Pro 2015 software (TreeAge Software, Inc, Williamstown, MA). Institutional review board (IRB) approval was not required as no human subjects were included. The model accounted for uncomplicated nulliparous women with non-anomalous singleton, vertex presenting fetuses who remained pregnant until 39 weeks gestation. Uncomplicated was defined based on the definition used by Gibson et al.[17] by excluding women with a prior uterine scar, planned or elective cesarean delivery, or with chronic maternal conditions that may lead to indicated delivery, including diabetes mellitus, chronic hypertension, cardiovascular disease, placental previa, or HIV positive status. As depicted in Fig 1, the two cohorts of the decision analytic tree were mutually exclusive, consisting of:

1. **elective induction** of labor at 39 weeks gestational age (eIOL), or
2. **expectant management** with induction of labor at 41 weeks if undelivered (EM).

The elective induction cohort was modeled to result in one of two delivery outcomes: vaginal or cesarean delivery. Operative vaginal deliveries were not included in this model as input probabilities were limited by data sources. Mode of delivery outcomes among the expectant management cohort were modeled in the following manner: at 39 weeks the mother may undergo spontaneous labor, or may require a medically or obstetrically indicated induction of labor; if the patient was undelivered at week 40 she may undergo spontaneous labor or she may develop a medical or obstetrical indication to undergo an induction of labor; if undelivered at 41 weeks she may undergo induction of labor and either have a successful vaginal delivery or be delivered by cesarean section.

Cesarean delivery rates among nulliparous women with a non-anomalous singleton, vertex presenting fetus were derived from data published by Gibson et al. [17] from an analysis of the Consortium on Safe Labor (CSL) and then confirmed in written communication in January 2017 (written confirmation available upon request). The CSL was funded by the National Institutes of Health with several goals, one of which was to “describe contemporary labor progression at the national level.” Trained staff reviewed over a quarter of a million deliveries at 19 hospitals in the United States from 2002 to 2008.[18] Using the CSL data allowed two key variables of this model to be populated with reliable, contemporary and generalizable data: a) CSL included information on inception cohort (n = 233,736 women) followed from gestational week 37 to 40. This enabled us to accurately calculate both numerator and denominator for the outcome of interest such as cesarean delivery, and b) modified Bishop score (characterized in a dichotomous fashion as favorable versus unfavorable) at the time of presentation to labor and delivery as it related to mode of delivery. For this model we used a modified Bishop score compromising dilation, effacement, and station to determine cervical ripeness. We defined an unripe cervix as a modified Bishop score ≤ 4 which was based on the manuscript by Gibson et al.[17] which was based on a prior publication from the CSL database.[19] One aspect of this variable not reported by the CSL was cesarean delivery rate as a function of Bishop score at 41 weeks. Therefore, data were analyzed and a curve fitting data projected the cesarean delivery rates as a function of Bishop score at 41 weeks (see S1 Fig). When data were missing and / or unavailable, probabilities were taken from contemporary literature as noted in S1 Appendix. When data did not exist, we analyzed existing data and a curve fitting the data was calculated (approximation).

The model accounted for these possible maternal outcomes: a mother with no delivery complications; a mother with morbidity defined as requiring blood transfusion, chorioamnionitis or preeclampsia; severe morbidity managed in labor and delivery including eclampsia, hemorrhage, uterine rupture, septic shock, unplanned hysterectomy, unplanned operation, or thromboembolic events; severe morbidity requiring management in an intensive care unit; or maternal death.

Regarding fetal outcomes there were two possibilities: the fetus survives until delivery or the fetus experiences an intrauterine fetal demise. If the fetus survives until delivery, the model accounted for the following neonatal outcomes: an infant without complications; an infant with morbidity including meconium stained amniotic fluid or a shoulder dystocia without lasting neurological damage; an infant with severe morbidity defined as a composite of major comorbidities (birth injuries, sepsis, pneumonia, intraventricular hemorrhage, aspiration, hypoxic ischemic encephalopathy, respiratory distress syndrome, seizures, oliguria, myocardial injury, ventilator use, CPAP use, transient tachypnea of the newborn, transfusions, or surfactant use), or a composite of respiratory morbidities (oxygen use, CPAP use, transient tachypnea of the newborn, or surfactant administration); or a neonatal death.

### Maternal and perinatal variables and baseline model assumptions

Baseline model assumptions were derived from the literature, primarily Gibson et al.’s analysis of CSL. We performed systematic reviews of the literature searching for all existing systematic reviews, randomized trials, registries and observational studies addressing the question of elective induction of labor at 39 versus expectant management with induction of labor at 41 weeks of gestational age. Because we found no empirical or modeling study directly answering this question, we proceeded with development of the model as described in this paper.

The model consisted of 41 variables, 101 evidenced-based estimates and 14 derived calculations. Collectively, they aimed to describe the probabilities of entering a particular health state and the morbidity and mortality associated with each of these states. S1 Appendix describes all variables used in the model.

Data used to inform the model were based on both randomized and observational studies; expert opinions were used to estimate the additional effect of the expected range of values/preferences. The most challenging aspect of this analysis was the question of how to account for the relative value associated with maternal versus. infant health. Since preferences of the unborn fetus are impossible to obtain, we approached this problem as follows: first, as customary, we assigned the value of 1 to perfect health status, 0 to death. To weigh the importance of maternal versus perinatal mortality but to ensure that the value for given health status can range between 0 and 1 only, we used the weights 1/(1+x) and x/(1+x) for mother and baby, respectively. Note that **1/(1+x) + x/(1+x) = 1;** x represents the ratio of importance of mother’s over infant’s health. This means if x = 2, health of mother is as twice as important; if x = 1/2, infant’s health is twice as important. This can also be thought as regret: how many times one would regret having a mother worse off than baby.[20] Similarly, we introduced variables yB and yM to represent the ratio of morbidity/mortality for infant and mother, respectively. For example, y = 1/4 mortality indicates that mortality was 4 times more important than morbidity. However, some morbidities can be more severe than the others. The morbidity for the newborn was weighted using the five minute Apgar score derived from National Vital Statistics reports [21] in such a way that categorized it as severe morbidity if the Apgar score was 1 to 3, non-severe if the score was 4 to 7, while a healthy baby was assigned Apgar score 8 to 10. We acknowledge the limitation of using Apgar scores as a marker for neonatal morbidity as infants with low Apgar scores can have normal acid-base status and vice versa [22] as well as the arbitrary Apgar cutoffs that define severe, non-severe and healthy. However, every infant born in the United States is assigned Apgar scores making it the only available health proxy available for all neonates.

For maternal health states, we relied on the systematic review by the Agency for Healthcare Research and Quality [23] to assign distribution of the utilities ranging from 0.6 to 1, which we then weighted by severe and non-severe morbidities and further categorized according to the management in labor and delivery or no Intensive Care Unit (ICU) versus ICU management. Because morbidity is disutility (*i*.*e*., the higher value refers to worse health status), non-ICU morbidity is lower than ICU morbidity and we arbitrary assigned the ICU morbidity to 0.5 = best, [24] 1 = worst, while non-severe morbidity was weighted as 0.3 = best and 1 = worst.

### Main analyses

The goal of the model was to simulate individual patient outcomes based on the existing data from the literature. Each pregnant woman moves through the health states according to random numbers drawn from given probability distributions. Because the event rates for key outcomes such as mortality is low, we repeated the process for a minimum of 100,000 patients (up to 1,000,000 women *i*.*e*., until we obtained saturation in the findings beyond which the increase number of simulations did not affect modeling results. The microsimulation allows generation of mean values with variations around the mean (*e*.*g*., standard deviation) for the entire group and the various subgroups of patients for the given outcomes of interest (*e*.*g*., maternal vs. infant mortality, etc.). The best strategy is the one which generated the lower values in terms of morbidity and mortality. The main results were expressed in terms of the frequency of the strategy generating the most “optimal outcomes” when the probability of the event is weighted with health state utility (ranging from 0 to 1- see below). The secondary analyses assessed the frequency of maternal and infant mortality, effect of the mode of delivery on the frequency of Cesarean deliveries, and the effect of morbidity and values and preferences on the choice of the optimal strategy. Note that we decided against presenting our main analyses as quality-adjusted life expectancy as some authors did in assessing the impact of delivery in 41 vs. 42 weeks;[25] quality-adjusted life expectancy reporting would require taking perspective of the mother. However, health of the baby is also important, and we took this into consideration as outlined above.

### Sensitivity analyses

Monte-Carlo microsimulation simultaneously samples each model parameter from a given probability distribution, which allows assessment of the effect of all parameters on the robustness of the model results. Nevertheless, we supplemented these multivariable analyses with one way Monte Carlo analyses of key variables of interest, allowing us to vary over the ranges that extend beyond the most plausible values specified in the multivariable analysis. We were particularly interested in the effect of maternal age, preferences / values, and morbidities–the latter initially specified based on our estimates. As explained above, Monte Carlo microsimulation allows sampling and assessment of uncertainties of the effects of management strategies by computing the analysis thousands of times by randomly drawing the numbers according to pre-specified probability distributions (see S1 Appendix). In turn, this allows hypothesis testing of the observed differences between the management strategies. Consequently, where appropriate, we tested for the differences in outcomes between two strategies (*e*.*g*., using chi-square test to assess differences between maternal, stillbirth, or perinatal mortality). S1 Appendix shows all variables and assumed distribution. Distributions were selected based on empirical evidence (normal distribution for age), *beta* (which represents a natural choice for representing uncertainty of the probability parameter where data describing that parameter are binomial—such as presence or absence of outcome of interest),[26] and triangular (where we could not find empirical data and relied on our best estimates, such as weighting morbidities).

## Results

**Fig 2:**
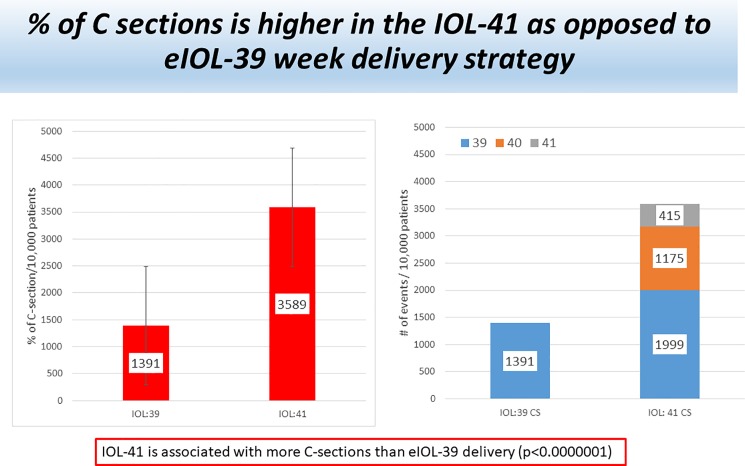
Lower cesarean delivery rates among eIOL as compared to EM. Expectant management resutled in significantly higher cesarean delivery rates as compared to the elective induction of labor strategy: 35.9% versus 13.9%, p<0.01.

Using a decision analytic model informed by contemporaneous literature, we found that elective induction of labor among uncomplicated nulliparous women with vertex presenting singleton fetuses resulted in less maternal and neonatal risk as compared to expectant management with induction of labor for medical or obstetrical indications or by 41 weeks if undelivered. The cesarean section rate was significantly higher in the expectant management cohort as compared to elective induction of labor as shown in Fig 2: 35.9% versus 13.9%, p<0.01. When patients with an unfavorable cervix were analyzed, 39 week eIOL still resulted in fewer cesarean deliveries as compared to EM (8.0% versus 26.1%, p<0.01).

Modeling 100,000 deliveries resulted in statistically similar rates of maternal mortality (0 maternal deaths in the elective induction of labor arm as compared to 1 maternal death in the expectant management arm, p = 0.32). However, there was a significantly higher rate of maternal morbidity among the expectant management group as compared to the elective induction of labor arm: 21.2% versus 16.5%, p<0.01.

More stillbirths occurred in the expectant management group: 0.13% versus 0%, p<0.01. Furthermore, there were more neonatal deaths among the expectant management group as compared to the elective induction cohort: 0.25% versus 0.12%, p< 0.03. Additionally, there were more infants with severe complications in the expectant management group as compared to the elective induction of labor group: 12.1% versus 9.4%, p<0.01.

Preference regarding the importance of maternal and perinatal health is an important factor. When the ratio of the importance of each member of the dyad was studied as a function of combined maternal and perinatal morbidity and mortality, elective induction of labor at 39 weeks was always the best strategy regardless of the preference of maternal vis a vis perinatal health. Similarly, elective induction at 39 weeks was the leading strategy when evaluating perinatal as compared to maternal mortality. Additionally, when evaluating the impact of maternal age and preferences for maternal morbidity as compared to mortality, again, elective induction at 39 weeks prevailed over expectant management.

## Discussion

Using Monte Carlo microsimulation methodology, we found that, among uncomplicated, nulliparous, non-anomalous, singleton, vertex pregnancies, elective induction of labor at 39 weeks resulted in less maternal and neonatal risk as compared to expectant management with subsequent induction of labor by 41 weeks gestation. Specifically, irrespective of cervical examination, the 39 week elective induction cohort experienced fewer cesarean deliveries, lower rates of maternal morbidity, fewer stillbirths and neonatal deaths, and less neonatal morbidity than mothers expectantly managed until 41 weeks.

While obstetric mantra from past eras stressed the increased risk of cesarean delivery with induction of labor, our findings–driven by reliable, contemporary and generalizable data from the Consortium of Safe Labor–complement contemporary literature that elective induction of labor actually decreases the risk of cesarean delivery. [27–29] Additionally, these results align with findings from a recent systematic review of randomized trials that elective induction does not increase cesarean rates.[16]

Importantly, our findings are also supported by biologically plausible explanations. Placental insufficiency has been proposed as an underlying mechanism for many otherwise unexplained, term stillbirths.[30] This diagnosis can explain why stillbirths increase with advancing gestational age in a stochastic fashion. Induction of labor at 39 weeks may lower rates of stillbirth as the placenta is still able to perfuse the fetus both prior to and during labor. Cord accidents are likely stochastic as well–increasing with in utero exposure and perhaps a dilated cervix. A rising occurrence of placental insufficiency could also contribute to an increase in cesarean deliveries due to non-reassuring fetal testing with increasing gestational age.[31]

Conversely, fetal weight also progressively increases with increasing gestational age. From week 39 to 41 fetal weight increases on average 100 to 150 grams per week.[32] Thus, the risk of macrosomia with its attendant effects on labor progress and the diagnosis of cephalopelvic disproportion, the leading indication for cesarean delivery, increases with increasing gestational age.[33] Similarly, macrosomia is a major risk factor for shoulder dystocia with permanent brachial plexus injury, a major source of serious neonatal morbidity.[34] Thus, the likelihood of cesarean delivery and serious neonatal morbidity should also display a stochastic relationship to duration of gestation. Together, deteriorating placental function and excess fetal growth provide strong biological plausibility to our findings that elective induction at 39 weeks reduces the risk of these adverse pregnancy outcomes.

While this study found no difference in rates of maternal mortality between the groups, maternal morbidity was significantly higher among the expectant management group. One reason many providers are opposed to elective induction of labor is attributed to the notion of increased rates of cesarean deliveries, and thus an increase in maternal morbidity. However, the opposite was found. Thus, a primary argument against elective induction at 39 weeks is without empirical support. Given the recent trends towards increased maternal morbidity and mortality,[35] elective induction of labor could be considered as one factor in comprehensive strategy to improve maternal outcomes.

Our study has several strengths. First, the rigorous Monte Carlo microsimulation methodology strengthens the results and our conclusions. This methodology allows sampling and assessment of uncertainties of the effects of management strategies. In turn, this allows hypothesis testing of the observed differences between the management strategies. Second, while some outcomes in our model are so rare that gaps in the literature do not address them, similarities between published literature and our study strengthens the reliability of our findings. We note that there is an ongoing, NIH-funded, randomized controlled trial randomizing uncomplicated, nulliparous patients with singleton, non-anomalous fetuses to either induction of labor at 39 weeks or expectant management. The primary outcome is a composite of severe perinatal morbidity and mortality (NCT01990612). We anxiously await the results of this significant study.

While our study has several strengths, there are also important limitations to address. First, all models, including ours, are based on information that vary in quality. Data used in the model were based mostly on observational studies which are inherently prone to bias. We attempted to overcome this bias in four ways: 1) by using data from the Consortium on Safe Labor which meticulously reviewed over a quarter of a million deliveries at nearly 20 centers in the United States; 2) when CSL data was missing or unavailable, we performed systematic searches of the literature to derive the best possible estimates for our model, 3) by performing Monte Carlo multivariable microsimulation to allow sampling of the large population based on the best and most plausible empirical estimated and theoretical assumptions, and 4) by performing sensitivity analyses examining effects of values and preferences on outcomes extending beyond the assumptions in the multivariable Monte Carlo Analysis. We also used clinical acumen to identify the most relevant outcomes and not necessarily all possible clinical outcomes that were reported in the literature. We believe that focusing on the most important outcomes generated more salient results, the significance of which could have been diluted by including outcomes that are admittedly clinically less relevant, or exceedingly rare. The consistency of the findings in all the analyses we performed increases the credibility of our analysis. However, despite these attempts, bias may not be completely eliminated. In addition, variations in obstetric management could also affect outcomes. While our study and others [36, 37] have found an increase in the rate of intrauterine fetal demise rate among patients in the expectant management group, it is unlikely that this population was undergoing antenatal fetal surveillance which would not normally be initiated until 40 to 41 weeks gestation. It is unknown if initiation of antenatal fetal surveillance at 39 weeks gestational age would mitigate the disparity in fetal deaths between the groups. Conversely, failure of practitioners to allow an adequate trial of labor could also negate observed benefits of an elective induction of labor. Furthermore, we are not able to elucidate the impact of elective induction of labor on the burden of perineal trauma.

Another extremely important aspect of this subject matter that is outside of the scope of this paper is: what do women want? While our results show less maternal and neonatal risk among women undergoing elective induction of labor at 39 weeks, the rates of these complications are still quite rare. Shared decision making is one of the cornerstones of modern medicine. Spontaneous labor without medical intervention is considered sacred by many women. Hospital admission for an induction of labor and the barrage of medical interventions is not the preference of many women. Moreover, the personal and societal costs of failed induction of labor are not directly accounted for in this model.

It should also be noted that it is unknown whether or not our healthcare system can adequately address the logistics of routine elective inductions at 39 weeks gestation, and if so, how these clinical findings translate into healthcare costs. Inductions of labor are time-intensive [38, 39] and can be costly; therefore, it is unclear if a policy of elective induction of labor at 39 weeks would be cost-effective. However, a preliminary estimate of cost savings appears supportive. If a policy of elective induction at 39 weeks gestational age saves 21,980 cesarean sections per 100,000 deliveries, and if a cesarean delivery costs roughly $3,900 USD more than a vaginal delivery, [40] one can realize potential financial savings associated with a strategy that can decrease cesarean rates to this degree. We acknowledge that a proper cost-effectiveness analysis may yield different results once all costs–including the additional healthcare costs for extended inductions–are accounted for; however, the potential savings does warrant further investigation.

In closing, our Monte Carlo microsimulation model demonstrates that elective induction of labor among uncomplicated, nulliparous patients with singleton, vertex, non-anomalous fetuses results in lower maternal and neonatal risk as compared to expectant management. Likely attributable to subsequent deteriorating placental function or cord accidents, induction of labor at 39 weeks is associated with decreased rates of stillbirth and cesarean deliveries due to non-reassuring fetal testing. Reductions in cumulative fetal weight and the occurrence of macrosomia likely explained decreased cesarean delivery rates with elective induction at 39 weeks. Additionally, there appears to be no maternal benefit to expectant management. These factors also account for the observed reductions in neonatal and maternal morbidity among patients undergoing planned induction. While these findings appear to be reassuring for the population as a whole, we acknowledge that not all women nor their providers desire elective inductions and we recommend that the patient should be the final arbiter of the timing and mode of delivery after adequate counseling and informed consent.

## Supporting information

S1 Fig Curve fitting data projecting cesarean rates as function of Bishop at 41 weeks. This image depicts the curve fitting the data projecting the cesarean delivery rates as a function of Bishop score at 41 weeks gestational age.
(DOCX)

S1 Appendix Baseline probabilities used in analytic model. This appendix outlines the input probabilities used to perform this decision analysis.
(DOCX)
