# Supplementary material for: Elective induction of labor at 39 weeks among nulliparous women: The impact on maternal and neonatal risk
Source: PLoS One. 2018 Apr 25;13(4):e0193169. doi: 10.1371/journal.pone.0193169 (PMC5918610; doi:10.1371/journal.pone.0193169)
Supplement: S1 Fig — This image depicts the curve fitting the data projecting the cesarean delivery rates as a function of Bishop score at 41 weeks gestational age. (DOCX) [file pone.0193169.s001.docx]

**Supplemental Figure prepared for:**

***Should all mothers with uncomplicated pregnancies undergo elective induction of labor at 39 weeks?***

Supplemental Figure 1: When data did not exist to inform the cesarean section rates as a function of Bishop score at 41 weeks gestational age, data from the Consortium of Safe Labor were analyzed and a curve fitting the data was calculated.
