# Supplementary material for: Elective induction of labor at 39 weeks among nulliparous women: The impact on maternal and neonatal risk
Source: PLoS One. 2018 Apr 25;13(4):e0193169. doi: 10.1371/journal.pone.0193169 (PMC5918610; doi:10.1371/journal.pone.0193169)
Supplement: S1 Appendix — This appendix outlines the input probabilities used to perform this decision analysis. (DOCX) [file pone.0193169.s002.docx]

**S1 Appendix: Baseline probabilities used in analytic model**

| ***Variable*** |  |  |  | |  | |  |  |  |
| --- | --- | --- | --- | --- | --- | --- | --- | --- | --- |
| ***General*** | | *Distribution* | *Mean* | *SD* |  |  | |  | ***Ref.***** |
| *Age* | | *Normal* | *28.63* | *6.18* |  |  | |  | *Matthews et al., 2015* |
| *Unfavorable Bishop Scores by week* | |  |  |  |  |  | |  |  |
| *Week 39* | | *Table* | *0.3937* |  |  |  | |  |  |
| *Week 40* | | *Table* | *0.4229* |  |  |  | |  |  |
| *Week 41* | | *Table* | *0.5158* |  |  |  | |  |  |
|  | | ***Elective IOL*** | | | ***Expectant Management*** | | | | ***Ref.*** |
| ***Probability*** | | *Distribution* | *Mean* | *SD* | *Distribution* | *Mean* | | *SD* |  |
| *Probability of C-section* | |  |  |  |  |  | |  |  |
| *Week 39* | | *Beta* | *0.01131* | *5.1 E-6* |  | *0.166868* | | *1.76 E-5* |  |
| *Week 40* | |  |  |  | *Beta* | *0.335451* | | *3.88 E-5* |  |
| *Week 41* | | *Beta* | *0.047541* | *6.28 E-5* |  | *0.40201* | | *1.44 E-4* |  |
| *Probability of spontaneous delivery* | |  |  |  |  |  | |  |  |
| *Week 39* | |  | *0.3206* | *0.00224* |  | *0.3206* | | *0.00224* |  |
| *Week 40* | | *Beta* | *0.4804* | *0.00318* | *Beta* | *0.4804* | | *0.00318* |  |
| *Week 41* | |  | *0.7972* | *0.00487* |  | *0.7972* | | *0.00487* |  |
| *Favorable* | |  |  |  |  |  | |  |  |
| *Week 39* | |  | *0.002958* | *0.00224* |  | *0.055141* | | *0.00224* |  |
| *Week 40* | | *Beta* | *0.008163* | *0.00318* | *Beta* | *0.126961* | | *0.00318* |  |
| *Week 41* | |  | *0.009620* | *0.00487* |  | *0.151138* | | *0.00487* |  |
| *Unfavorable* | |  |  |  |  |  | |  |  |
| *Week 39* | |  | *0.0083518* | *0.00224* |  | *0.111727* | | *0.00224* |  |
| *Week 40* | | *Beta* | *0.0338999* | *0.00318* | *Beta* | *0.208489* | | *0.00318* |  |
| *Week 41* | |  | *0.0378917* | *0.00487* |  | *0.250997* | | *0.00487* |  |
|  | | ***Elective IOL*** | | | ***Expectant Management*** | | | | ***Ref.*** |
| ***Mortality*** | | *Distribution* | *Mean* | *SD* | *Distribution* | *Mean* | | *SD* |  |
| *Maternal mortality (Favorable & Unfavorable)* | |  |  |  |  |  | |  |  |
| *Week 39* | | *Beta* | *2.0 E-9^(*)^* | *5.1 E-10* |  | *2.0 E-9^(*)^* | | *5.1 E-10* |  |
| *Week 40* | |  |  |  | *Beta* | *2.0 E-9^(*)^* | | *5.1 E-10* |  |
| *Week 41* | |  |  |  |  | *2.0 E-9^(*)^* | | *5.1 E-10* |  |
| *Maternal mortality (C-section) (Favorable & Unfavorable)* | |  |  |  |  |  | |  |  |
| *Week 39* | | *Beta* | *2.0 E-9^(*)^* | *5.1 E-10* |  | *2.0 E-9^(*)^* | | *5.1 E-10* |  |
| *Week 40* | |  |  |  | *Beta* | *2.0 E-9^(*)^* | | *5.1 E-10* |  |
| *Week 41* | |  |  |  |  | *2.0 E-9^(*)^* | | *5.1 E-10* |  |
|  | |  |  |  |  |  | |  |  |
| *Table 1*  ***Variable*** *(Continued)* | |  |  |  |  |  | |  |  |
|  | | ***Elective IOL*** | | | ***Expectant Management*** | | | | ***Ref.*** |
| ***Mortality*** | | *Distribution* | *Mean* | *SD* | *Distribution* | *Mean* | | *SD* |  |
| *Fetal mortality (stillbirths) (Favorable)* | |  |  |  |  |  | |  |  |
| *Week 39* | | *Beta* | *2.0 E-9^(*)^* | *5.1 E-10* |  | *0.001128* | | *3.5 E-4* |  |
| *Week 40* | |  |  |  | *Beta* | *0.001343* | | *4.2 E-4* |  |
| *Week 41* | |  |  |  |  | *0.002188* | | *1.4 E-3* |  |
| *Fetal mortality (stillbirths) (Unfavorable)* | |  |  |  |  |  | |  |  |
| *Week 39* | | *Beta* | *2.0 E-9^(*)^* | *5.1 E-10* |  | *0.002385* | | *7.9 E-4* |  |
| *Week 40* | |  |  |  | *Beta* | *0.001924* | | *7.7 E-4* |  |
| *Week 41* | |  |  |  |  | *0.000931* | | *1.3 E-3* |  |
| *Fetal mortality (stillbirths) (C-section) (Favorable)* | |  |  |  |  |  | |  |  |
| *Week 39* | | *Beta* | *2.0 E-9^(*)^* | *5.1 E-10* |  | *0.000688* | | *9.6 E-4* |  |
| *Week 40* | |  |  |  | *Beta* | *0.000554* | | *7.7 E-4* |  |
| *Week 41* | |  |  |  |  | *2.0 E-9^(*)^* | | *5.1 E-10* |  |
| *Fetal mortality (stillbirths) (C-section) (Unfavorable)* | |  |  |  |  |  | |  |  |
| *Week 39* | | *Beta* | *2.0 E-9^(*)^* | *5.1 E-10* |  | *0.002091* | | *1.16 E-3* |  |
| *Week 40* | |  |  |  | *Beta* | *0.000921* | | *7.9 E-4* |  |
| *Week 41* | |  |  |  |  | *0.000942* | | *1.3 E-3* |  |
| *Neonatal mortality (Favorable)* | |  |  |  |  |  | |  |  |
| *Week 39* | | *Beta* | *2.0 E-9^(*)^* | *5.1 E-10* |  | *0.002256* | | *4.9 E-4* |  |
| *Week 40* | |  |  |  | *Beta* | *0.001343* | | *4.2 E-4* |  |
| *Week 41* | |  |  |  |  | *0.000728* | | *1.01 E-3* |  |
| *Neonatal mortality (Unfavorable)* | |  |  |  |  |  | |  |  |
| *Week 39* | | *Beta* | *0.002174* | *3.01 E-3* |  | *0.001908* | | *5.88 E-4* |  |
| *Week 40* | |  |  |  | *Beta* | *0.003848* | | *1.06 E-3* |  |
| *Week 41* | |  |  |  |  | *0.002793* | | *1.84 E-3* |  |
| *Neonatal mortality (C-section) (Favorable)* | |  |  |  |  |  | |  |  |
| *Week 39* | | *Beta* | *2.0 E-9^(*)^* | *5.1 E-10* |  | *0.005502* | | *2.05 E-3* |  |
| *Week 40* | |  |  |  | *Beta* | *0.003326* | | *1.46 E-3* |  |
| *Week 41* | |  |  |  |  | *0.00369* | | *3.15 E-3* |  |
| *Neonatal mortality (C-section) (Unfavorable)* | |  |  |  |  |  | |  |  |
| *Week 39* | | *Beta* | *0.006993* | *0.01372* |  | *0.002091* | | *5.88 E-4* |  |
| *Week 40* | |  |  |  | *Beta* | *0.004146* | | *1.45 E-3* |  |
| *Week 41* | |  |  |  |  | *0.003763* | | *2.08 E-3* |  |
|  | |  |  |  |  |  | |  |  |
| *Table 1*  ***Variable*** *(Continued)* | |  |  |  |  |  | |  |  |
|  | | ***Elective IOL*** | | | ***Expectant Management*** | | | | ***Ref.*** |
| ***Morbidities*** | | *Distribution* | *Mean* | *SD* | *Distribution* | *Mean* | | *SD* |  |
| *Probability of non-severe baby’s complications (Favorable)*  *(RESP complications; Shoulder dystocia)* | |  |  |  |  |  | |  |  |
| *Week 39* | | *Beta* | *0.020023* | *3.39 E-3* |  | *0.020551* | | *1.02 E-3* |  |
| *Week 40* | |  |  |  | *Beta* | *0.020873* | | *1.12 E-3* |  |
| *Week 41* | |  |  |  |  | *0.025293* | | *3.02 E-3* |  |
| *Probability of non-severe baby’s complications (Unfavorable) (RESP complications; Shoulder dystocia)* | |  |  |  |  |  | |  |  |
| *Week 39* | | *Beta* | *0.012141* | *3.78 E-3* |  | *0.024877* | | *1.73 E-3* |  |
| *Week 40* | |  |  |  | *Beta* | *0.027778* | | *1.96 E-3* |  |
| *Week 41* | |  |  |  |  | *0.037639* | | *4.12 E-3* |  |
| *Probability of non-severe baby’s complications (C-section)*  *(Favorable) (RESP complications; Shoulder dystocia)* | |  |  |  |  |  | |  |  |
| *Week 39* | | *Beta* | *0.019231* | *1.24 E-2* |  | *0.028581* | | *3.1 E-3* |  |
| *Week 40* | |  |  |  | *Beta* | *0.025562* | | *2.66 E-3* |  |
| *Week 41* | |  |  |  |  | *0.025783* | | *4.87 E-3* |  |
| *Probability of non-severe baby’s complications (C-section)*  *(Unfavorable) (RESP complications; Shoulder dystocia)* | |  |  |  |  |  | |  |  |
| *Week 39* | | *Beta* | *0.028269* | *1.03 E-2* |  | *0.023656* | | *2.5 E-3* |  |
| *Week 40* | |  |  |  | *Beta* | *0.025059* | | *2.4 E-3* |  |
| *Week 41* | |  |  |  |  | *0.037159* | | *4.12 E-3* |  |
| *Probability of severe baby’s complications (Favorable)* | |  |  |  |  |  | |  |  |
| *Week 39* | | *Beta* | *0.058153* | *7.95 E-3* |  | *0.068916* | | *2.57 E-3* |  |
| *Week 40* | |  |  |  | *Beta* | *0.078257* | | *2.97 E-3* |  |
| *Week 41* | |  |  |  |  | *0.092633* | | *7.84 E-3* |  |
| *Probability of severe baby’s complications (Unfavorable)* | |  |  |  |  |  | |  |  |
| *Week 39* | | *Beta* | *0.071739* | *1.21 E-2* |  | *0.076318* | | *4.1 E-3* |  |
| *Week 40* | |  |  |  | *Beta* | *0.089885* | | *4.74 E-3* |  |
| *Week 41* | |  |  |  |  | *0.125257* | | *1.06 E-2* |  |
| *Probability of severe baby’s complications (C-section) (Favorable)* | |  |  |  |  |  | |  |  |
| *Week 39* | | *Beta* | *0.102564* | *3.48 E-2* |  | *0.132737* | | *8.9 E-3* |  |
| *Week 40* | |  |  |  | *Beta* | *0.115854* | | *7.54 E-3* |  |
| *Week 41* | |  |  |  |  | *0.140481* | | *1.49 E-2* |  |
| *Table 1*  ***Variable*** *(Continued)* | |  |  |  |  |  | |  |  |
|  | | ***Elective IOL*** | | | ***Expectant Management*** | | | | ***Ref.*** |
| ***Morbidities*** | | *Distribution* | *Mean* | *SD* | *Distribution* | *Mean* | | *SD* |  |
| *Probability of severe baby’s complications (C-section) (Unfavorable)* | |  |  |  |  |  | |  |  |
| *Week 39* | | *Beta* | *0.118881* | *2.72 E-2* |  | *0.10873* | | *7.1 E-3* |  |
| *Week 40* | |  |  |  | *Beta* | *0.117918* | | *2.09 E-2* |  |
| *Week 41* | |  |  |  |  | *0.161017* | | *1.13 E-2* |  |
| *Probability of non-severe maternal complications (Favorable)*  *(Lacerations or Episiotomies + Infectious complications)* | |  |  |  |  |  | |  |  |
| *Week 39* | | *Beta* | *0.082042* | *6.58 E-3* |  | *0.124428* | | *2.48 E-3* |  |
| *Week 40* | |  |  |  | *Beta* | *0.139146* | | *2.85 E-3* |  |
| *Week 41* | |  |  |  |  | *0.193078* | | *8.42 E-3* |  |
| *Probability of non-severe maternal complications (Unfavorable)*  *(Lacerations or Episiotomies + Infectious complications)* | |  |  |  |  |  | |  |  |
| *Week 39* | | *Beta* | *0.1* | *1.00 E-3* |  | *0.12002* | | *3.68 E-3* |  |
| *Week 40* | |  |  |  | *Beta* | *0.136527* | | *4.20 E-3* |  |
| *Week 41* | |  |  |  |  | *0.204233* | | *9.27 E-3* |  |
| *Probability of non-severe maternal complications (C-section) (Favorable) (Lacerations or Episiotomies + Infectious complications)* | |  |  |  |  |  | |  |  |
| *Week 39* | | *Beta* | *0.058065* | *1.93 E-2* |  | *0.143239* | | *6.85 E-3* |  |
| *Week 40* | |  |  |  | *Beta* | *0.164818* | | *6.51 E-3* |  |
| *Week 41* | |  |  |  |  | *0.154584* | | *1.18 E-2* |  |
| *Probability of non-severe maternal complications (C-section) (Unfavorable) (Lacerations or Episiotomies + Infectious complications)* | |  |  |  |  |  | |  |  |
| *Week 39* | | *Beta* | *0.095745* | *1.76 E-2* |  | *0.130804* | | *5.67 E-3* |  |
| *Week 40* | |  |  |  | *Beta* | *0.135896* | | *5.43 E-3* |  |
| *Week 41* | |  |  |  |  | *0.168737* | | *8.52 E-3* |  |
| *Probability of ICU admission due to severe maternal complications* | |  |  |  |  |  | |  |  |
| *Week 39* | | *Beta* | *0.000468* | *0.000022448* |  | *0.000468* | | *0.000022448* | *ACOG Practice Bulletin 146* |
| *Week 40* | |  |  |  | *Beta* | *0.00044* | | *0.000022959* | *ACOG Practice Bulletin 146* |
| *Week 41* | |  |  |  |  | *0.00050* | | *0.000035969* | *ACOG Practice Bulletin 146* |
| *Probability of ICU admission due to severe maternal complications*  *(C-section)* | |  |  |  |  |  | |  |  |
| *Week 39* | | *Beta* | *0.00174* | *0.000071938* |  | *0.00174* | | *0.000071938* | *ACOG Practice Bulletin 146* |
| *Week 40* | |  |  |  | *Beta* | *0.00181* | | *0.000075510* | *ACOG Practice Bulletin 146* |
| *Week 41* | |  |  |  |  | *0.00174* | | *0.000100765* | *ACOG Practice Bulletin 146* |
| *Table 1*  ***Variable*** *(Continued)* | |  |  |  |  |  | |  |  |
|  | | ***Elective IOL*** | | | ***Expectant Management*** | | | | ***Ref.*** |
| ***Morbidities*** | | *Distribution* | *Mean* | *SD* | *Distribution* | *Mean* | | *SD* |  |
| *Probability of maternal complications (non-ICU) (Favorable)* | |  |  |  |  |  | |  |  |
| *Week 39* | | *Beta* | *0.114355* | *1.11 E-2* |  | *0.075420* | | *3.25 E-3* |  |
| *Week 40* | |  |  |  | *Beta* | *0.082344* | | *3.78 E-3* |  |
| *Week 41* | |  |  |  |  | *0.107973* | | *1.27 E-2* |  |
| *Probability of maternal complications (non-ICU) (Unfavorable)* | |  |  |  |  |  | |  |  |
| *Week 39* | | *Beta* | *0.032178* | *9.02 E-3* |  | *0.078198* | | *5.10 E-3* |  |
| *Week 40* | |  |  |  | *Beta* | *0.084337* | | *5.77 E-3* |  |
| *Week 41* | |  |  |  |  | *0.084691* | | *1.13 E-2* |  |
| *Probability of maternal complications (non-ICU) (C-section) (Favorable)* | |  |  |  |  |  | |  |  |
| *Week 39* | | *Beta* | *0.112903* | *4.07 E-2* |  | *0.074116* | | *8.88 E-3* |  |
| *Week 40* | |  |  |  | *Beta* | *0.063267* | | *7.51 E-3* |  |
| *Week 41* | |  |  |  |  | *0.081911* | | *1.61 E-2* |  |
| *Probability of maternal complications (non-ICU) (C-section) (Unfavorable)* | |  |  |  |  |  | |  |  |
| *Week 39* | | *Beta* | *0.045872* | *2.04 E-2* |  | *0.074431* | | *7.79 E-3* |  |
| *Week 40* | |  |  |  | *Beta* | *0.077178* | | *7.71 E-3* |  |
| *Week 41* | |  |  |  |  | *0.059524* | | *9.83 E-3* |  |
| (*) Because software limitation, we imputed (2.0 E-9) in place of 0  ** Data derived from Gibson et al., 2014 unless stated otherwise. | |  |  |  |  |  | |  |  |
